# Magnetism and Thermal Transport of Exchange-Spring-Coupled La_2/3_Sr_1/3_MnO_3_/La_2_MnCoO_6_ Superlattices with Perpendicular Magnetic Anisotropy

**DOI:** 10.3390/nano13212897

**Published:** 2023-11-03

**Authors:** Vitaly Bruchmann-Bamberg, Isabell Weimer, Vladimir Roddatis, Ulrich Ross, Leonard Schüler, Karen P. Stroh, Vasily Moshnyaga

**Affiliations:** 1 Erstes Physikalisches Institut, Georg-August-University Göttingen, Friedrich-Hund-Platz 1, 37077 Göttingen, Germanyisabell.weimer@uni-goettingen.de (I.W.); leonard.schueler@uni-goettingen.de (L.S.);; 2 Helmholtz Centre Potsdam, GFZ German Research Centre for Geosciences, Telegrafenberg, 14473 Potsdam, Germany; vladimir.roddatis@gfz-potsdam.de; 3 IV. Physikalisches Institut, Georg-August-University Göttingen, Friedrich-Hund-Platz 1, 37077 Göttingen, Germany; uross@gwdg.de

**Keywords:** superlattices, high-resolution STEM, exchange spring magnet, perpendicular magnetic anisotropy, magneto-thermal conductivity

## Abstract

Superlattices (SLs) comprising layers of a soft ferromagnetic metal La_2/3_Sr_1/3_MnO_3_ (LSMO) with in-plane (IP) magnetic easy axis and a hard ferromagnetic insulator La_2_MnCoO_6_ (LMCO, out-of-plane anisotropy) were grown on SrTiO_3_ (100)(STO) substrates by a metalorganic aerosol deposition technique. Exchange spring magnetic (ESM) behavior between LSMO and LMCO, manifested by a spin reorientation transition of the LSMO layers towards perpendicular magnetic anisotropy below *T*_SR_ = 260 K, was observed. Further, 3ω measurements of the [(LMCO)_9_/(LSMO)_9_]_11_/STO(100) superlattices revealed extremely low values of the cross-plane thermal conductivity *κ*(300 K) = 0.32 Wm^−1^K^−1^. Additionally, the thermal conductivity shows a peculiar dependence on the applied IP magnetic field, either decreasing or increasing in accordance with the magnetic disorder induced by ESM. Furthermore, both positive and negative magnetoresistance were observed in the SL in the respective temperature regions due to the formation of 90°-Néel domain walls within the ESM, when applying IP magnetic fields. The results are discussed in the framework of electronic contribution to thermal conductivity originating from the LSMO layers.

## 1. Introduction

Mixed valence perovskite manganites with general chemical formula A_1−x_A′_x_MnO_3_ and double perovskites A_2_BB’O_6_ (here, A = La, Sr; B = Mn, Co) belong to the family of strongly correlated transition metal oxides. Their hallmark is a strong coupling between charge, spin, and lattice degrees of freedom, which results in super- and double-exchange (SE and DE) mechanisms of orbital interactions [1,2]. The variety of magnetic ordering, i.e., para-(PM), ferro-(FM), and anti-ferromagnetic (AFM) accompanied by metal-insulator and/or charge ordering transitions, can be effectively influenced via bandwidth or band-filling controls, i.e., by means of chemical pressure or hole doping. Moreover, the external control parameters, like temperature, hydrostatic pressure as well as applied magnetic and electric fields were found to strongly influence the phase transitions [3]. A unique field-induced phenomenon found in this material class is colossal magnetoresistance (CMR) or a magnetic field-induced insulator-metal transition [1,4,5]. Perovskite manganites have drawn great and continuous interest for basic and applied research in spintronics, multiferroics, catalysis, optoelectronics, and thermoelectricity [6,7,8,9,10].

Combining materials in superlattices (SL) has proven to be a fruitful pathway in the search for advanced functionality, leading to the discovery of a wide range of novel material properties not akin to the parent systems [11,12,13]. Magnetic SLs exhibit remarkable phenomena such as giant magnetoresistance (GMR) or tunnel magnetoresistance (TMR) and are nowadays widely used in sensing and information storage technologies. Perpendicular magnetic anisotropy (PMA) plays a crucial role in the increase of storage density in hard drives [14,15]. To achieve PMA in manganites, like LSMO, orbital and strain engineering approaches via the choice of appropriate substrates [16], buffer layers [17], or interfaces [18] have been suggested. These strategies are based on the promotion of a preferred occupation of the 3z^2^-r^2^ orbital, which favors PMA through spin-orbit coupling [19].

Another possibility to realize PMA in a soft FM with in-plane (IP) magnetic anisotropy is a direct-exchange coupling to a hard FM-possessing PMA [20]. Generally, exchange-coupled heterostructures or SLs of hard and soft FMs form the magnetic exchange spring (ESM) [21,22,23,24,25], which enables a gradual rotation or torsion of magnetic moments within the soft FM layers under applied small/moderate magnetic fields. The magnetic moments of the hard FM remain unchanged, thereby creating a spring-like reversible twist of the soft magnetic moments. This phenomenon occurs as long as the applied fields do not exceed the coercive field of the hard FM, ensuring its remanence. The interface spins of the soft FM remain effectively pinned by exchange coupling to the hard layers form a magnetic spring with a spin structure that resembles a domain wall [26,27]. It was shown that magnetoresistance (MR) can originate from the field-induced domain-wall-like twisted spin disorder in ESM NiFe/CoSm bilayers [26], and it was also found in intrinsic domain walls in LSMO nanowires [28].

However, knowledge of the electronic and thermal properties of ESM-coupled layers in manganite-based SLs is scarce [18]. Moreover, to the best of our knowledge, the thermal conductivity of ESM-coupled SLs and the influence of magnetic fields on it have not been studied. In general, thermal conductivity in isolating crystals is mainly based on phononic heat transport, which is affected by crystal symmetry, impurities, and other structural defects or distortions. The additional contribution of free-charge carriers to thermal conductivity in metals and highly doped semiconductors can be calculated using the Wiedemann-Franz (WF) law *κ*_el_ = *σ*_el_ × *L* × *T* [29], with electrical conductivity *σ*_el_, temperature *T*, and the Lorenz number *L* = *π*^2^/3(*k*_B_/*e*)^2^ = 2.45 *×* 10^−8^ Ω W/K^2^. However, in non-degenerate semiconductors as well as in strongly correlated or otherwise complex systems, the Lorenz number is not constant and can vary with temperature [30]. This makes the precise disentanglement of lattice and electronic contributions to thermal transport challenging. Still, the change of thermal conductivity in an external magnetic field, dubbed as “magneto-thermal conductivity” [31] and denoted as *MTC* = [*κ*(B ≠ 0) − *κ*(B = 0)]/*κ*(B = 0), can be observed in magnetoresistive materials due to the variation of their electrical conductivity dependent on a magnetic field [32,33,34,35,36,37].

Here we report an ESM coupling between LSMO and LMCO within [(LMCO)_n_/(LSMO)_n_]_m_ SLs epitaxially grown on STO(100) substrates via a metalorganic aerosol deposition (MAD) technique (see Methods section for details). Such an exchange spring with PMA, dictated by the hard magnetic LMCO layers, strongly impacts the electrical and thermal transport properties of SLs through magnetic field control of spin order/disorder. The results obtained highlight an attractive opportunity to study the spin-dependent scattering of charge carriers at domain-wall-hosting interfaces as well as to control the electromagnetic and thermal properties of SLs with a magnetic field.

## 2. Materials and Methods

### 2.1. Sample Preparation and Characterization

All oxide film samples were prepared by means of the metalorganic aerosol deposition (MAD) technique. Aerosols of the metalorganic precursor solution (acetylacetonates of the desired metals solved in N,N-dimethylformamide) have been sprayed through compressed air on a heated substrate. By using precise dosing units for liquid precursors as well as in situ growth control by means of optical ellipsometry [38], the single oxide films and heterostructures can be grown with monolayer accuracy. The substrate temperatures for growth of the crystalline oxide films were *T*_dep,cryst_ = 900–950 °C and the deposition rate was *v*_dep_ = 0.1 nm/s. For amorphous growth, a temperature of *T*_dep,amorph_ = 360 °C with a deposition rate of *v*_dep_ = 0.05 nm/s was used. The STO substrates (Crystal GmbH, 10 × 5 × 0.5 mm^3^) were TiO_2_ terminated based on an etching procedure [39] using an ammonium fluoride buffered hydrofluoric acid etchant and tempering for 1 h at *T* = 965 °C in the air to obtain a flat terrace morphology. The [(LMCO)_n_/(LSMO)_n_]_m_/STO(100) SLs were grown with an overall thickness of around d ≈ 75 nm by varying the superlattice repetition number “m”.

Four-probe electrical conductivity and magnetization measurements were carried out by Quantum Design GmbH (Darmstadt, Germany) PPMS and SQUID magnetometers MPMS XL and MPMS 3. The latter has been used for the rotator measurements (Standard rotator sample holder with α = 0° corresponding to the in-plane direction (α = 90° out-of-plane), samples were cut to 3 × 3 mm^2^ to fit the holder).

The heater (150 nm thick Au layer grown on 5 nm thick Cr adhesion layer) for thermal conductivity measurements by the 3ω method was deposited by thermal evaporation (Cr) and magnetron sputtering (Au) followed by structuring with an optical lithography lift-off (Karl SUSS MJB4 (SÜSS MicroTec, Garching bei München, Germany) exposure unit, Allresist AR-P 5350 photoresist (Micro Materials Pty Ltd., Malvern Victoria, Australia). The dimensions of the heater line (width 2b = 25 µm, the length between the voltage leads l ≈ 1 mm) were measured by optical microscopy and additionally confirmed by scanning electron microscopy. To electrically decouple the metal heater from the analyzed and potentially conductive samples, insulating capping layers of amorphous alumina (am-Al_2_O_3_) were used.

X-ray measurements were conducted with the Bruker Advance D8 (Ettlingen, Germany) diffractometer. X-ray reflectivity (XRR) was used to obtain the film thicknesses as well as the density of amorphous am-Al_2_O_3_ capping layers ρ_am-Al2O3_ = 3.3(2) g·cm^−3^ from single film measurements after fitting with the *GenX* program [40].

Scanning Transmission Electron Microscopy (STEM) was performed using a Thermo Fisher Scientific (TFS) (Thermo Fisher Scientific, Waltham, MA, USA) Themis Z 80-300 (S)TEM operated at 300 kV, equipped with a TFS SuperX Energy Dispersive X-ray (EDX) detector and a Gatan Imaging Filter (GIF) Continuum 1065. The microscope was tuned for a sub-Angstrom resolution with a beam convergence angle of 21.4 mrad. Specimens for STEM were prepared with a lift-out Focused Ion Beam technique using a TFS Helios G4UC dual-beam instrument. The octahedral tilt within the Mn-O layers was measured from iDPC-STEM images using the Atomap library for Python [41], by fitting 2D Gaussians to individual atom contrast features and evaluating the collection of atom positions regarding the tilt of the Mn-O bonds within the zone axis projection.

### 2.2. Measurements of Thermal Conductivity by the 3ω Method

The 3ω method is a well-established technique for measuring thermal conductivity, especially of thin films [42]. The home-built setup used here was built based on the original setup by Cahill. A metal stripe on top of the sample acts both as a heater and a thermometer. Sourcing AC (here using the Keithley 6221 (Tektronix UK Ltd., Berkshire, UK) current source) provides Joule heating of the metal line such that its temperature oscillates at a doubled frequency. Detecting the temperature oscillation via the calibrated temperature-dependent electrical resistance oscillation of the heater element is achieved by measuring the 3rd harmonic (3ω) AC voltage by lock-in amplification (here by Stanford Research Systems SR830, Sunnyvale, CA, USA). To suppress the dominant ohmic voltage drop, an in-situ hardware subtraction of a reference resistor with a low-temperature coefficient of resistance (Vishay Z201 foil resistor, Vishay Electronic GmbH, Selb, Germany) is realized by a voltage divider and instrumentation amplifiers (Texas Instruments INA103, Freising, Germany). The measured in-phase and out-of-phase 3ω voltage oscillations are then used to calculate the complex temperature oscillation of the metal heater.

The 3ω measurements have been carried out using the PPMS cryostat for temperature control. Continuous measurements of temperature-dependent temperature oscillations were performed at a cooling rate of 1 K/s with 3 s lock-in integration time at excitation angular frequencies of ln(ω) = 5.5 and 6, respectively.

Using an analytical expression for frequency-dependent temperature oscillation of a metal heater/thin film/substrate system derived by Borca-Tasciuc et al. [43], which was subsequently improved by the Olson, Graham, and Chen’s thermal impedance model [44], allows for fitting the data to obtain both thermal diffusivity and thermal conductivity of the substrate as well as the thermal resistance of a film on the substrate. Additional statistical evaluation for an estimation of the noise level of the raw 3-omega (voltage) signal has been also performed. Converting the signal to the temperature oscillation and subsequently calculating thermal resistance within the standard 3-omega evaluation procedure, results in an uncertainty of the thermal resistance of σ(R, thermal) = 2 × 10^−10^ m^2^K/W. Using the LSMO sample as an example, the error propagation leads to a statistical random error for thermal conductivity of σ(kappa) = 0.02 Wm^−1^K^−1^, which corresponds to a 2-sigma limit of ~2%.

## 3. Results and Discussion

### 3.1. Structure and Microstructure of LSMO/LMCO Superlattices

**Figure 1:**
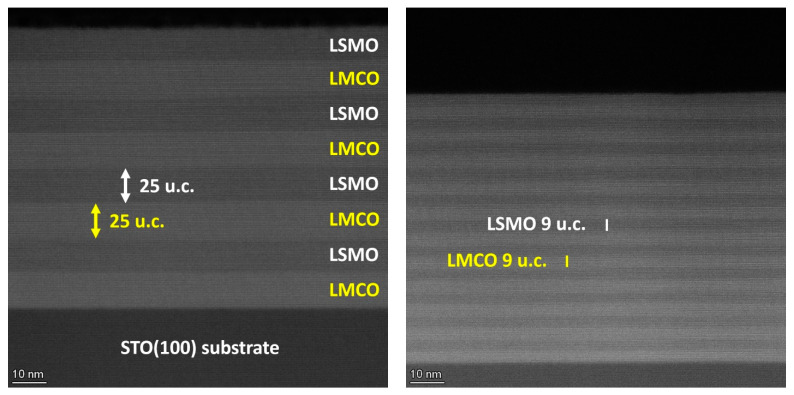
High angular annular dark field (HAADF) scanning transmission electron microscopy (STEM) images of the representative [(LMCO)_24_/(LSMO)_24_]_4_/STO(100) (**left**) and [(LMCO)_9_/(LSMO)_9_]_11_/STO(100) (**right**) SLs.

The SL samples composed of LMCO and LSMO, i.e., [(LMCO)_n_/(LSMO)_n_]_m_/STO(100), with layer thicknesses ranging from n = 1–24 unit cells (u.c.), were grown with SL repetition numbers m between 4 and 96 to ensure an overall thickness of d ≈ 75 nm for all samples. The structure and microstructure of the representative SLs with n = 9, 24 u.c. and m = 11, 4 are shown in Figure 1. The HAADF-STEM images reveal an epitaxial growth of LSMO and LMCO layers with regular repetition of the layers, each showing thicknesses close to the nominal ones. Moreover, the interfaces look sharp and flat in good agreement with the root-mean-square roughness of S_q_ ≈ 0.2 nm for the n = 9 u.c. SL and S_q_ ≈ 0.5 nm for the n = 24 u.c. SL, determined at the SL surfaces by means of atomic force microscopy (Figure S1, in Supplemental Material (SM). The small angle X-ray reflection (XRR) (see Figure S2 in SM) additionally confirms the thickness of individual layers to be close to the nominal values. Finally, X-ray diffraction (XRD) patterns evidence an out-of-plane epitaxy with c-axis lattice parameters within the range of *c* ≈ 0.3849–0.3855 nm for both LMCO and LSMO layers (see Figure S2, SM) This is not very surprising given the similarity in pseudocubic bulk lattice parameters of LMCO and LSMO, both having values close to c ≈ 0.388 nm [45,46] and sharing the same tensile stress state induced by the STO(100) substrate as well. One has to point out weak HAADF-STEM contrast between the LMCO and LSMO layers, having slightly differing composition at A-sites (La/La_0.7_Sr_0.3_) as well as very similar atomic masses of B-site cations, i.e., Co(59) and Mn(54). This makes the analysis of interfacial sharpness and intermixing at the atomic scale difficult. The additionally performed TEM chemical analysis of the [(LMCO)_9_/(LSMO)_9_]_11_/STO(100) SL by using energy dispersive X-ray microanalysis (EDX) (see Figure S3, SM) has revealed a clear chemical contrast between LMCO and LSMO as well as detected Co/Mn intermixing at the interfaces with a thickness ~2 u.c. This is in line with XRR results.

### 3.2. Magnetic Exchange Spring in LSMO/LMCO Superlattices

**Figure 2:**
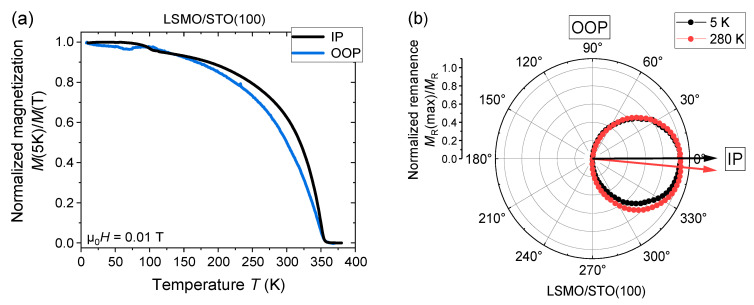

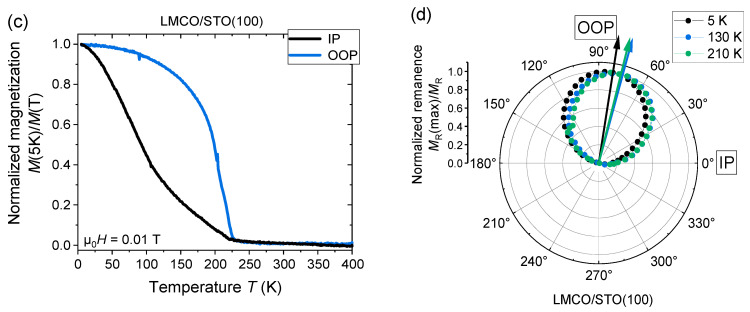
Normalized field-cooled IP and OOP magnetization of (**a**) LMCO/STO(100) and (**c**) LSMO/STO(100) single films with thickness d = 25 nm and 40 nm, respectively. The respective polar plots (**b**,**d**) show the angle-resolved measurements of the normalized remanent magnetization. The arrows indicate the position of the easy axis.

In order to elaborate on the magnetic properties of [(LMCO)_n_/(LSMO)_n_]_m_/STO(100) SLs, we first introduce single films of LSMO/STO(100) and LMCO/STO(100). The optimally doped perovskite manganite La_0.7_Sr_0.3_MnO_3_ (LSMO) is a well-known soft FM metal with an in-plane (IP) magnetic easy axis and magnetotransport properties governed by the DE interaction [47]. An almost 100% spin polarization at the Fermi level in the ground state [48] makes LSMO promising for spintronic applications. In Figure 2a, field-cooled IP and out-of-plane (OOP) magnetization measurements of a MAD-grown LSMO/STO(100) thin film with a thickness of d = 25 nm are shown, exhibiting a Curie temperature of *T*_C,LSMO_ = 355 K. Together with the rotator measurements of the remanent magnetization (Figure 2b) and measurements of magnetic hysteresis (Figure S4, SM), we conclude a soft FM behavior with an IP easy axis and a small coercive field µ_0_*H*_c_(5 K) ≈ 3 mT in agreement with previous reports on LSMO/STO(100) thin films [49].

The double perovskite La_2_MnCoO_6_ (LMCO) is an insulating hard FM characterized by an SE mechanism [45]. A MAD-grown LMCO/STO(100) film of d = 40 nm displays a high *T*_C_ = 225 K (Figure 2c) and possesses a large coercive field of µ_0_*H*_c_(5 K) ≈ 1.1 T (see Figure S3, SM). These values, being comparable to those measured in the B-site ordered bulk material [45] and previously studied MAD-grown LMCO thin films [50,51], evidence a right cation stoichiometry and an absence of oxygen deficiency, which is known to suppress magnetism in rf-sputtered LMCO films [52]. In addition, our LMCO/STO(100) film possesses an OOP magnetic easy axis as verified by the angle-dependent measurements of remanent magnetization shown in Figure 2d. This observation agrees well with previous reports on LMCO/STO(100) films [53,54]. Small deviations of the easy axes from the pure IP or OOP orientations in the presented measurements can either be explained by the competition between the crystal (OOP) and shape (thin film, IP) anisotropies or, most probably, they originate from an error of angle settings (here typically ±5°).

**Figure 3:**
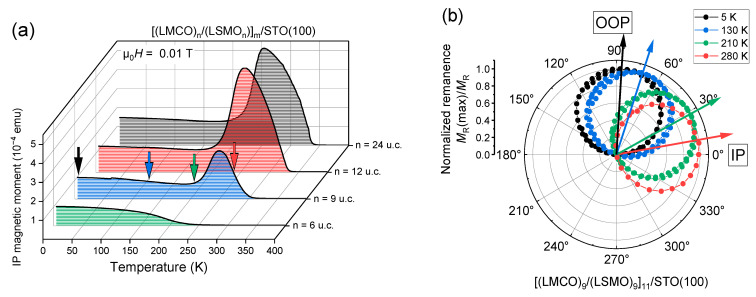
(**a**) Field-cooled IP magnetic moment component of [(LMCO)_n_/(LSMO)_n_]_m_/STO(100) SLs with different layer thicknesses n = 6, 9, 12 and 24 u.c. and bilayer numbers m = 16, 11, 8, 4, respectively. The arrows mark the temperatures used for the rotator measurements of normalized remanence of the n = 9 u.c. [(LMCO)_9_/(LSMO)_9_]_11_/STO(100) SL (**b**), showing a change of magnetic anisotropy from an OOP to an IP direction around 250 K upon warming from 5 K to 280 K. Here the arrows indicate the apparent position of the easy axis.

Figure 3a presents measurements of the IP magnetic moment of selected SLs, revealing an unusual magnetic behavior. Namely, the SLs with n = 9–24 u.c. show a spin reorientation (SR) transition at *T*_SR_ ≈ 260 K, at which the magnetic easy axis gradually changes towards PMA at low temperatures as evidenced by the temperature-dependent angle-resolved measurements of remanent magnetization, shown in Figure 3b representatively for the SL with n = 9 u.c. SLs with very thin layers n = 1–6 u.c. (see Figure S5, SM) do not reveal the SR transition, which is probably caused by two reasons: (1) very thin LSMO layers become “magnetically dead”, implying a significant reduction of *T*_C,LSMO_ < 200 K when the LSMO thickness is reduced down to few unit cells [55,56,57] and (2) the Co/Mn intermixing (see Figure S3, SM) in very thin layers n = 1–3 u.c. leads to the formation of a mixed (La,Sr)(Co,Mn)O_3_ with an unknown composition.

A similar magnetic PMA spin reorientation has been reported in heterostructures of perovskite LSMO and brown-millerite LaCoO_2.5_ (B-LCO), i.e., [(LSMO)_i_/(B-LCO)_j_]_m_/STO(100) heterostructures, by Zhang et al. [18]. They interpreted the SR based on the symmetry-mismatch-driven perovskite/brown-millerite interfacial elongation of the oxygen octahedra, which implies an orbital reconstruction of the Mn ions within the interfacial LSMO. Such reconstruction was suggested to change the magnetic easy axis of LSMO towards a PMA geometry. The magnetic contribution of the B-LCO was neglected due to the very low Curie temperature of a single B-LCO/STO(100) film, *T*_C,B-LCO_ << *T*_SR_. Thus, they attributed the PMA observed in their SLs to the structural and orbital reconstruction, induced by the symmetry breaking at the perovskite/brown-millerite interface.

**Figure 4:**
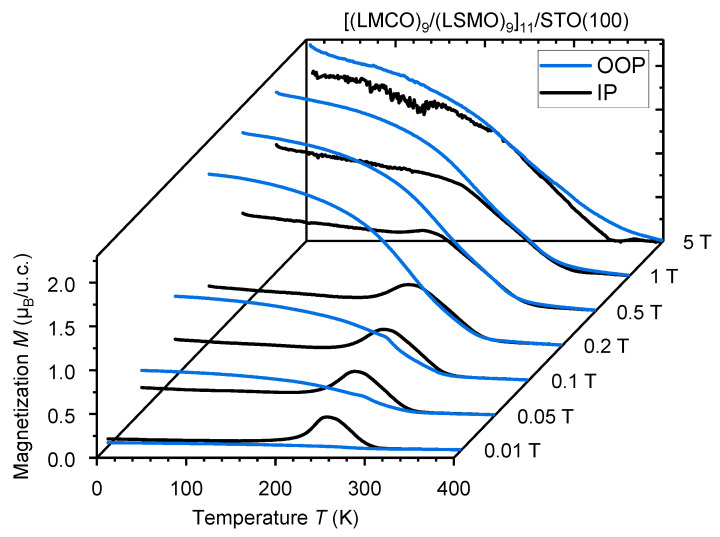
IP and OOP field-cooled magnetization measurements of the n = 9 u.c. [(LMCO)_9_/(LSMO)_9_]_11_/STO(100) SL in different magnetic fields.

We have carried out measurements of both IP and OOP field-cooled magnetization under various external magnetic fields (Figure 4) on the SL with n = 9 u.c., which has the highest interface density among the samples that exhibit the SR transition. A similar behavior to that reported by Zhang et al. [18] has been observed, i.e., (1) IP spin reorientation for T < T_SR_ ≈ 260 K; (2) saturation of the OOP magnetization at low temperatures for cooling fields *B* ≥ 0.2 T; and (3) an IP-OOP crossover at lower fields and temperatures around 260 K. Note, that in contrast to Zhang et al. [18], our [(LMCO)_n_/(LSMO)_n_]_m_/STO(100) SLs possess a perovskite/perovskite heteroepitaxy without any structural symmetry mismatch at the interface as evidenced by high-resolution HAADF-STEM and iDPC-TEM measurements (see Figure S6 in SM). Evidently, the interfaces appear coherent and show no visible abrupt changes in the octahedral tilt/rotation angles.

According to our magnetization measurements, the observed spin reorientation in our SLs can more likely be explained by the interplay of different magnetic easy axes and strongly different coercive fields of LMCO and LSMO, leading to the formation of a “magnetic exchange spring” (ESM)-coupled heterostructure. The reduction of the IP magnetization of our SLs below *T*_SR_ would then be attributed to the OOP rotation of magnetic moments within the LSMO due to the exchange coupling to the hard magnetic LMCO with PMA. This would indeed require a slightly increased *T*_C_ of either the LMCO layer itself or, at minimum, its interfacial region near the layer LSMO, up to a value of *T*_C,SL-LMCO_ ≈ *T*_SR_ ≈ 260 K within the SLs. An independent method for estimating the T_C_ of LMCO layers in SLs is provided by Raman spectroscopy measurements, which allow for measuring the spin-spin correlation induced anomalous shift of Raman lines in FM manganites due to spin-phonon coupling [58]. As one can see in Figure S7 (SM), the anomalous downshift of the dominating LMCO Raman line around 600 cm^−1^, induced by the FM transition, indeed starts at T_C_ ≈ 260 K, which is significantly larger than the *T*_C_ = 225 K of single LMCO films determined also by Raman spectroscopy [50]. Thus, thin (9–24 u.c.) LMCO layers in LMCO/LSMO SLs do have an enhanced *T*_C_ compared to single LMCO/STO films likely because of the reduced dimensionality and optimal interfacing with FM metallic LSMO layers.

**Figure 5:**
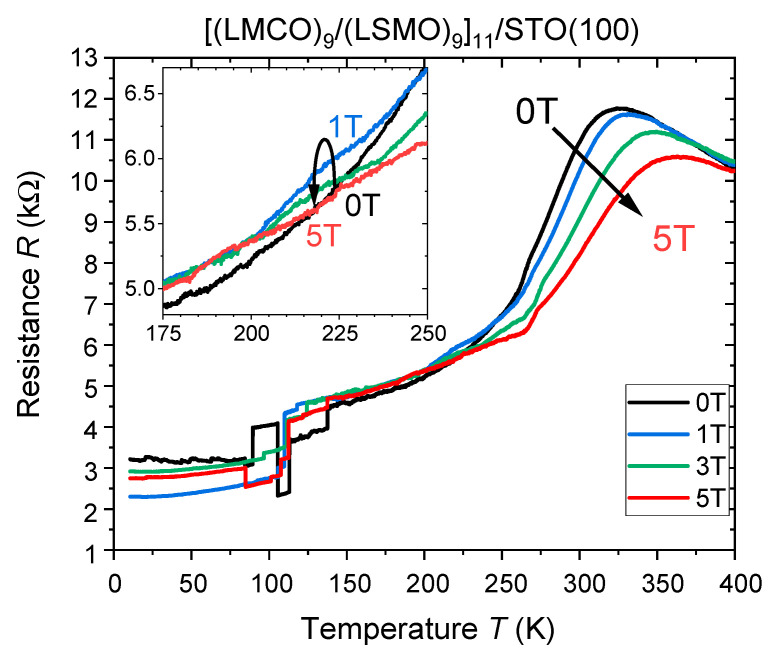
Field-cooled electrical resistance vs. temperature of the [(LMCO)_9_/(LSMO)_9_]_11_/STO(100) SL with both current and field applied in-plane. The inset shows the region with positive magnetoresistance.

Due to the orthogonality of the easy axes of the LSMO and LMCO layers, the formation of 90° Néel-type domain walls at the interfaces between the layers at low temperatures is favored. Such ESM made from the insulating LMCO and metallic/magnetoresistive LSMO counterparts should certainly give rise to a unique field-dependent electrical resistivity behavior. Temperature- and field-dependent electrical resistance measurements (current IP, field-cooled) of the n = 9 u.c. SL are shown in Figure 5.

One can see a metal-insulator transition with a peak temperature around *T*_MI_ ≈ 320 K for *B* = 0 T and a maximal value of *CMR*(300 K) = −36%. This can be attributed solely to the LSMO, as current flow takes place only within the metallic LSMO. A similar *R*(*T*) behavior is known for single LSMO/STO(100) films of comparable thickness and is reported elsewhere [59]. Below *T* < 250 K, especially in the region around *T* ≈ 220 K, the resistance of the SL shows an increase with the magnetic field and magnetoresistance becomes positive *MR*(220K, *B* = 1 T) = +4.5 % for 0 < *B* < 5 T (see inset in Figure 5). This is in clear contradiction with the well-known negative CMR effect. Note, that at *T*_SR_ = 260 K, the spin reorientation and the ESM formation set in, resulting in the field-induced spin disorder within the emergent Néel domain-wall, which is expected to increase the electron-spin scattering and thus the resistance of the LSMO. When nearly saturated (*B* ≥ 5 T), the spin disorder vanishes as all spins are oriented IP, and the LSMO within the SL again follows its intrinsic negative CMR behavior.

Further, a strong decrease of the resistance below *T* ≈ 275 K as well as the apparent stochastic resistance jumps below *T* < 100–150 K can be seen in Figure 5. The former can be caused by the mutual ferromagnetic proximity, as the magnetic moment of the adjacent LMCO is also the source of an additional magnetic field and thereby able to cause a reduction of LSMO resistance due to CMR. The other aspect of resistance jumps at lower temperatures might be ascribed to domain-wall pinning and movement as well as creation or annihilation of magnetic domain wall boundaries within the ultrathin LSMO in field-cooled measurements, which is known to alter the resistance of nanoscale LSMO [28]. Upon warming, however, the resistance does not show any jumps, most likely indicating domain rotation rather than domain wall movement (see Figure S8, SM).

**Figure 6:**
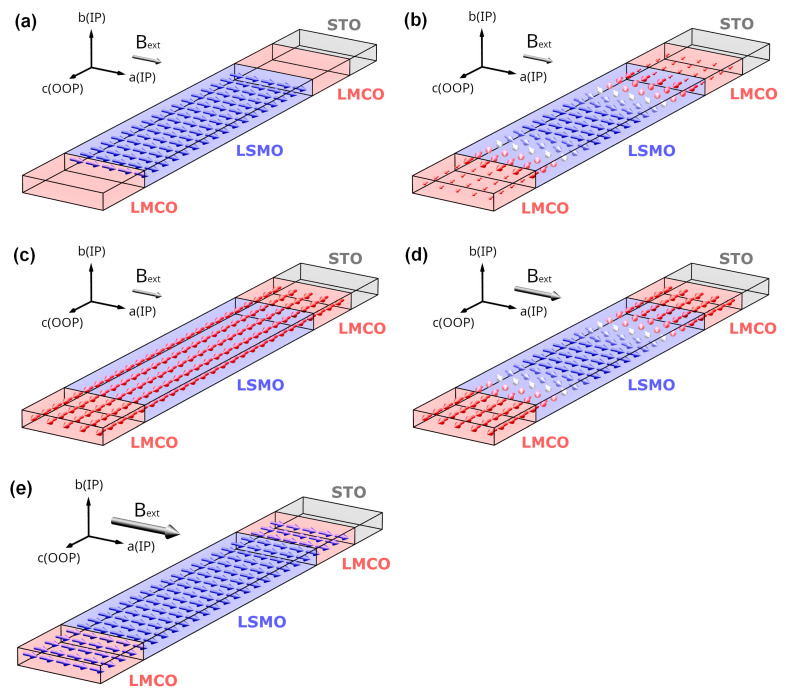
Schematic illustrations of the temperature- and magnetic field-dependent magnetic profile within an ESM in [(LMCO)_n_/(LSMO)_n_]_m_/STO(100) SLs: (**a**) T_SR_ < T < T_C, LSMO_ (**b**) PMA of the constituent layers leading to the formation of 90° Néel domain walls for T < T_SR_ and low IP fields; (**c**) for T << T_SR_ and low IP fields the IP magnetic moment is vanishingly small, and (**d**) reorientation of LSMO spins back to IP for T << T_SR_ and increasing IP field; (**e**) Model for SLs in the saturated state, at high IP magnetic fields exceeding the H_C_ of LMCO.

The observations mentioned above allow us to model the typical profile of a magnetic exchange spring within the LMCO/LSMO SL as sketched in Figure 6. At temperatures *T*_C,LMCO_ < *T* < *T*_C_,_LSMO_, the magnetic moments of the LSMO layers lie along their respective IP easy axis direction (Figure 6a). As the temperature falls below *T* < *T*_C,LMCO_ and the LMCO becomes OOP ferromagnetic, the exchange coupling at the interface forces the LSMO to rotate OOP, thus favoring Néel-type domain walls at the interfaces (Figure 6b). By increasing the IP magnetic field, the magnetic moments of the LSMO reorient back to the IP direction (d), followed by saturation of the entire SL when the applied magnetic field exceeds the coercive field of the LMCO (e).

### 3.3. Magneto-Thermal Conductivity of LSMO/LMCO SL

**Figure 7:**
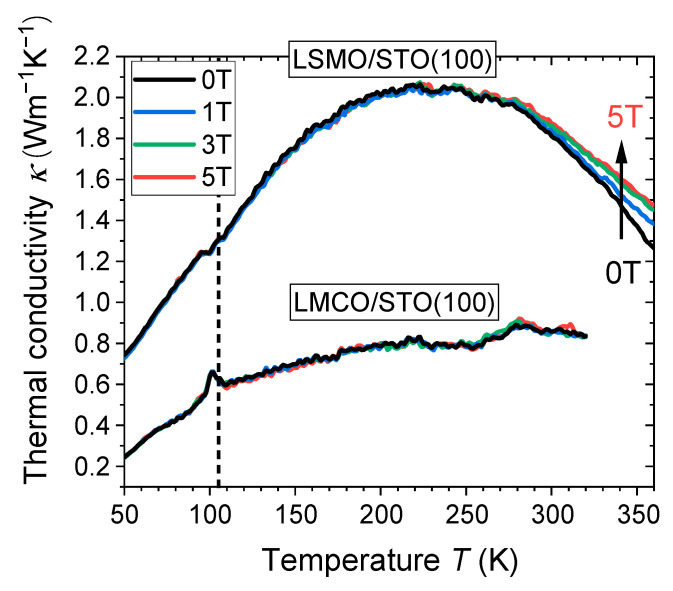
Temperature dependencies of the field-cooled cross-plane thermal conductivity of LSMO/STO(100) and LMCO/STO(100) single films with the thicknesses *d* = 100 nm and 25 nm, respectively, in applied in-plane magnetic fields, *B* = 0–5 T. The dashed line marks the position of the cubic-tetragonal transition of the STO substrate, *T*_STO_ = 105 K.

To analyze the temperature and magnetic field behavior of thermal conductivity *κ*(*T*, *B*) in LSMO/LMCO SLs, we first measured the cross-plane thermal conductivity of the constituting single films LSMO/STO(100) and LMCO/STO(100). The data on *κ*(*T*, *B*) are shown in Figure 7. One can see that, throughout the measured temperature range, the thermal conductivity of the LSMO film either remains unchanged or increases under an applied magnetic field. This can be quantified by a positive magneto-thermal conductivity (MTC), which can be as large as *MTC*(360 K) = 100% × (*κ*(B) − *κ*(0))/*κ*(0) = +17% at *B* = 5T. This value is definitely outside of the 2σ limit of ~2% (see Methods), making the *MTC* = 17% reasonable. Note that this MTC is confined to a relatively narrow temperature window close to *T*_C,LSMO_ and seems to correlate with the CMR effect: Positive MTC values can be explained by a CMR-related field-induced increase of electrical conductivity in the LSMO film thereby increasing the electronic contribution to thermal conductivity and thus enabling MTC. However, an estimation of the pure electronic part of thermal conductivity by using the WF law accounts only for a small fraction of the total measured MTC (see Figure S8, ref. [37]). Note, that the WF law, more or less satisfactorily describing the behavior of conventional metals and failing already for more complex semiconducting [30] and nanocrystalline metallic [60] systems, must not be applicable for strongly correlated electron systems, i.e., LSMO. This probably indicates that other possible magnetic field-induced changes of (a) heat capacity; (b) magnons; (c) oxygen octahedral tilt angles, and (d) Jahn-Teller disorder [33,61] might contribute to MTC, but they are difficult to disentangle into separate contributions.

Double perovskite LMCO films on STO(100) with a monoclinic structure possess a higher degree of octahedral tilting (Mn-O-Co angle of φ_LMCO_ = 154° (ref. [54]), as compared to that in rhombohedral LSMO/STO(100) (Mn-O-Mn angle φ_LSMO_ = 168° (ref. [56]). In addition, the insulating behavior of the cation-ordered LMCO due to superexchange and the doubled unit cell led to a significantly lower thermal conductivity compared to that in LSMO (Figure 7). Further, no influence of magnetic field on thermal transport in LMCO is observable, which fits the absence of the CMR effect in Co/Mn-ordered double perovskites [62]. Finally, one can see in Figure 7 a clear peak in the thermal conductivity of LMCO around *T* = 105 K, i.e., close to the temperature of the structural (cubic-tetragonal) phase transition of the STO substrate. Considering the epitaxial character of LMCO/STO films and strong elastic coupling to the substrate, this peak in *κ*(105 K) can be ascribed to a change in the LMCO strain state due to the structural phase transition in the STO substrate.

**Figure 8:**
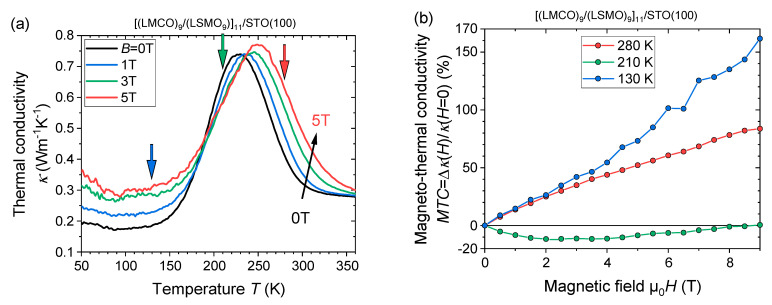
(**a**) Field-cooled cross-plane thermal conductivity of the [(LMCO)_9_/(LSMO)_9_]_11_/STO(100) superlattice in different applied in-plane magnetic fields. The arrows mark the temperatures used for the field-dependent magneto-thermal conductivity (MTC). (**b**) Field-dependent MTC of the [(LMCO_9_)/(LSMO)_9_]_11_/STO(100) superlattice.

The temperature and magnetic field dependence of the cross-plane thermal conductivity of the [(LMCO_9_)/(LSMO)_9_]_11_/STO(100) SL is presented in Figure 8. Remarkably ultralow thermal conductivity *κ*_SL_(300 K) = 0.32 Wm^−1^K^−1^ has been obtained in this SL sample in zero field. Similar values were reported in the literature for bulk samples of all-inorganic vacancy-ordered double perovskites (e.g., *κ*_Cs2SnI6_ (295 K) = 0.29 Wm^−1^K^−1^ (ref. [63]), Ruddlesden-Popper perovskites (e.g., *κ*_Cs2PbI2Cl2_ (295 K) = 0.37 Wm^−1^K^−1^ (ref. [64]), or chalcogenides like Ag_2_Se (*κ*_Ag2Se_ (300 K) = 0.29 Wm^−1^K^−1^ (ref. [65]).

Considering the SL geometry with thermal resistances of LMCO and LSMO connected in series, the ultralow cross-plane thermal conductivity of the SL could, in principle, originate both from the LMCO and LSMO layers as well as from the LSMO/LMCO interfaces. Note, that both the LMCO and LSMO layers in the SL, being only 9 u.c. thick, could be much less thermally conducting than the LSMO and LMCO single films in Figure 7. This is in line with the reduction and variation of oxygen octahedral rotation tilt angles determined from high-resolution iDPC images of the [(LMCO_9_)/(LSMO)_9_]_11_/STO(100) SL shown in Figure S5 (SM). The obtained values for the Mn-O-Mn(Co) angles φ vary in the range of approximately 154° < φ < 160°. Within the LMCO layers, the angles center at φ_SL-LMCO_ ≈ 154° which is similar to bulk, while within the LSMO layers, the values reach φ_SL-LSMO_ ≈ 160°, having a stronger tilt compared to the bulk LSMO with φ_LSMO_ ≈ 166° (ref. [46]). In between, the values smoothly change across the interfaces. We suspect that the observed modulations of the octahedral tilt angles along the growth direction and the stronger tilting of LSMO oxygen octahedra in the SL compared to bulk LSMO could be the origin of the ultralow thermal conductivity in the LSMO/LMCO SLs.

Two important features appear when applying an external magnetic field (Figure 8): (1) A shift of the thermal conductivity peak to higher temperatures and (2) the emergence of an unexpected negative MTC within the temperature region 185 K < T < 235 K (see Figure 8a). The shift of the thermal conductivity peak of the SL in applied fields towards higher temperatures can likely be explained by the shift of the magnetic (*T*_C_) and electric (*T*_MI_) transition temperatures towards higher values, which is common for perovskite FM manganites [66]. However, neither of the single manganite films has revealed a negative MTC. The field-dependent MTC of the n = 9 u.c. SL is shown in Figure 8b for selected temperatures. One can see a minimum negative MTC reaching as low as *MTC*(210 K, 3 T) ≈ −11%. For stronger fields of 4 T < *B* < 9 T, the amplitude of the negative MTC decreases and even almost changes sign for *B* = 9 T. A possible explanation for the observed behavior is the field-induced spin disorder within the LSMO layers promoted by the ESM formation. The spin disorder affects the charge carrier mobility due to enhanced spin scattering (cf. Figure 5d) and could thus result in a reduction of the electronic contributions to the thermal conductivity of the SL.

## 4. Conclusions

Superlattices of La_2/3_Sr_1/3_MnO_3_/La_2_CoMnO_6_ (LSMO/LMCO) were epitaxially grown on SrTiO_3_(100) substrates using the metalorganic aerosol deposition technique. Their magnetic behavior was shown to be governed by perpendicular magnetic anisotropy, originating from magnetically hard double perovskite LMCO layers. An exchange-spring magnetic coupling between LSMO and LMCO layers was observed, leading to a spin reorientation transition of LSMO at *T*_SR_ = 260 K. Applied in-plane magnetic fields enable control of the degree of spin disorder through continuous spin rotation within the soft magnetic LSMO layers towards their natural in-plane easy axis. Such field-controlled spin order/disorder was found to strongly influence both electrical and thermal transport in the SLs via spin-dependent scattering of charge carriers and their contributions to thermal transport in LSMO. The results obtained highlight the suitability of exchange-spring magnetic coupling within perovskite superlattices not only for achieving perpendicular magnetic anisotropy with nanoscale layer thicknesses down to ~9 u.c. (~3.5 nm) but also for controlling both electrical resistance and thermal transport using magnetic fields.

## Data Availability

The data presented in this study are available on request from the corresponding author.

## Supplementary Materials

The following supporting information can be downloaded at: https://www.mdpi.com/article/10.3390/nano13212897/s1. Figure S1: Left: Atomic force microscope topography of the [(LMCO)_n_/(LSMO_n_)]_m_/STO(100) n = 9 u.c. SL (RMS roughness S_q_ = 0.2(1) nm); Right: SL n = 24 u.c. (S_q_ = 0.5(1) nm; Figure S2: Left: XRR patterns (black curves) and fitting curves using the program *GenX3* [40] of [LSMO_n_/LMCO_n_]_m_/STO(100) SLs revealing superstructure peaks of the bilayers. The fit parameters are listed in Table S1. Right: X-ray diffraction patterns of the SLs reveal a very similar out-of-plane lattice parameter *c* ≈ 3.852 ± 0.004 Å for all SLs and single LSMO and LMCO films. The arrows indicate the k_α1_/k_α2_ spliting of the STO(200) peak and of the (002) peak of most SLs; Table S1: *GenX*3 fit parameters for [LSMO_n_/LMCO_n_]_m_/STO(100) SLs; Figure S3: Distribution of Co (top, left) and Mn (top, right) atoms within the [(LMCO)_9_(LSMO)_9_]_11_ SL obtained by using energy dispersive X-ray microanalysis (EDX) (left panels) shows a clear chemical contrast between the LMCO and the LSMO layers. A linear scan along the growth direction of the whole SL (bottom, left) and along the selected layers in the middle of SL (bottom, right), from which a (Co/Mn) intermixing at the LSMO/LMCO interfaces with a thickness ~2 u.c. can be deduced; Figure S4: Magnetic hysteresis loops for LSMO/STO(100) (left) and LMCO/STO(100) (right) at 5K along their respective easy axis; Table S2: Saturation magnetization and coercive field of LSMO/STO(100) and LMCO/STO(100) along their easy axis; Figure S5: Left: Field-cooled IP magnetic moment of the [(LMCO)_n_/(LSMO_n_)]_m_/STO(100) (n = 1–9 u.c.) superlattices at low magnetic field. Spin reorientation transition is missing in SLs with very thin LSMO and LMCO layers. Right: IP Magnetic hysteresis loops of selected [(LMCO)_n_/(LSMO_n_)]_m_/STO(100) superlattices; Figure S6: ADF-STEM (top, left), iDPC (top, right) images of the [LSMO_9_/LMCO_9_]_11_/STO(100) superlattice, with the corresponding evaluation of octahedral tilt angles θ within the image plane (bottom). Connection to the Mn-O-Mn(Co) angle in the main text is done via φ_B-O-B_ = 180° − 2θ. The central cluster around θ = 0° (on the right panel) corresponds to the Mn-Mn pairs, while the datapoints around θ = ±12° correspond to the Mn-O-Mn(Co) bond chains. The analysis reveals continuous change of the octahedral tilt angle at the interfaces between LMCO (θ_LMCO_ ≈ 13°) and LSMO (θ_LSMO_ ≈ 10°). Error bars indicate the maximum scattering of the angles within one layer, while the standard deviation for all layers lies in the range of σ(θ) = 0.5–1.5°; Figure S7: Left: Raman spectra of the n = 24 u.c. SL [(LSMO)_24_/(LMCO)_24_]_4_/STO(100) measured for different temperatures in the range *T* ≈ 100–400 K and an illustration of the 644 cm^−1^ breathing mode vibration; Right: Temperature dependence of the position of the breathing line (left scale) and IP magnetic moment (right scale) indicates a significant increase of the Curie temperature of the LMCO layers within the SL up to about *T*_C,LMCO_ ≈ 260 K. The red line is a fit to the anharmonic line shift $\omega (T)=\omega_{0}+C\left(1+2/\left(e^{\hbar \omega_{0}/{2k}_{B}T}-1\right)\right)$ [67] for temperatures $T>T_{C,LMCO}$; Figure S8: Electrical resistance vs. temperature (both current and magnetic field applied in-plane) in the vicinity of structural phase transition in STO at T* = 105 K for the [(LMCO)_9_/(LSMO_9_)]_11_/STO(100) SL at different magnetic fields. The arrows indicate the direction of the temperature scan; Figure S9: Top panel: measured magneto-thermal conductivity, MTC = 100% × [κ(5 T) − κ(0)]/κ(0), (black points) and the estimated from the Wiedemann-Franz (WF) law for electronic contricbution to MTC due to CMR (red points) of the single LSMO/STO(100). Bottom panel: colossal magnetoresistance CMR = 100% × [R(0) − R(5 T)]/R(0) of the LSMO/STO(100) thin film shows a typical behaviour with a peak close to *T*_C_ ≈ 350 K. References [40,67] are cited in the Supplementary Materials.
