# Supplementary material for: Magnetism and Thermal Transport of Exchange-Spring-Coupled La2/3Sr1/3MnO3/La2MnCoO6 Superlattices with Perpendicular Magnetic Anisotropy
Source: Nanomaterials (Basel). 2023 Nov 3;13(21):2897. doi: 10.3390/nano13212897 (PMC10649329; doi:10.3390/nano13212897)
Supplement: Supplementary file 1 [file nanomaterials-13-02897-s001.zip › nanomaterials-2665233-supplementary.pdf]

Supplemental Material

Magnetism and Thermal Transport of Exchange-Spring-Coupled $La_{2/3}Sr_{1/3}MnO_3/La_2MnCoO_6$ Superlattices with Perpendicular Magnetic Anisotropy

Vitaly Bruchmann-Bamberg[1], Isabell Weimer[1], Vladimir Roddatis[2], Ulrich Ross[3], Leonard Schüler[1], Karen P. Stroh[1], Vasily Moshnyaga[1]

[1]Erstes Physikalisches Institut, Georg-August-University Göttingen, Friedrich-Hund-Platz 1, 37077 Göttingen, Germany

[2]Helmholtz Centre Potsdam, GFZ German Research Centre for Geosciences, Telegrafenberg, 14473 Potsdam, Germany

[3]IV. Physikalisches Institut, Georg-August-University Göttingen, Friedrich-Hund-Platz 1, 37077 Göttingen, Germany

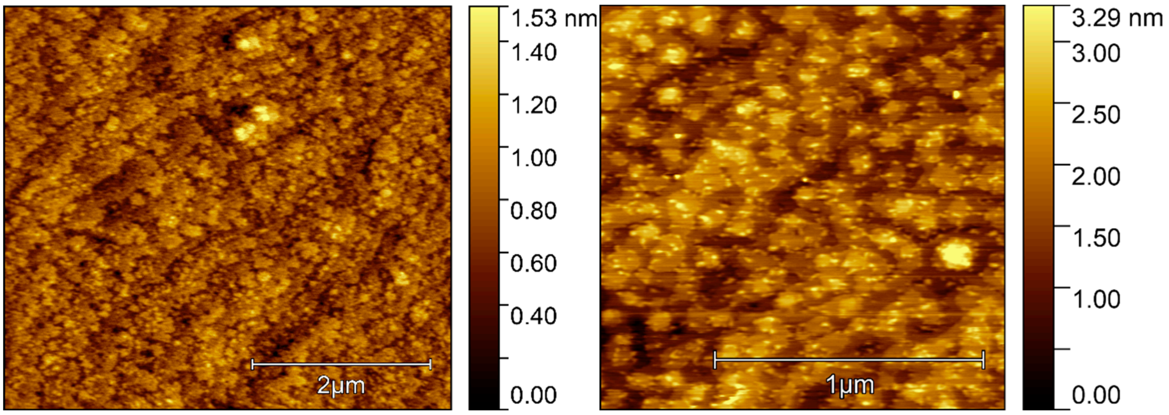

Figure S1 Left: Atomic force microscope topography of the $[(LMCO)_n/(LSMO_n)]_m$/STO(100) n=9 u.c. SL (RMS roughness S_q=0.2(1) nm); Right: SL n=24 u.c. (S_q=0.5(1) nm)

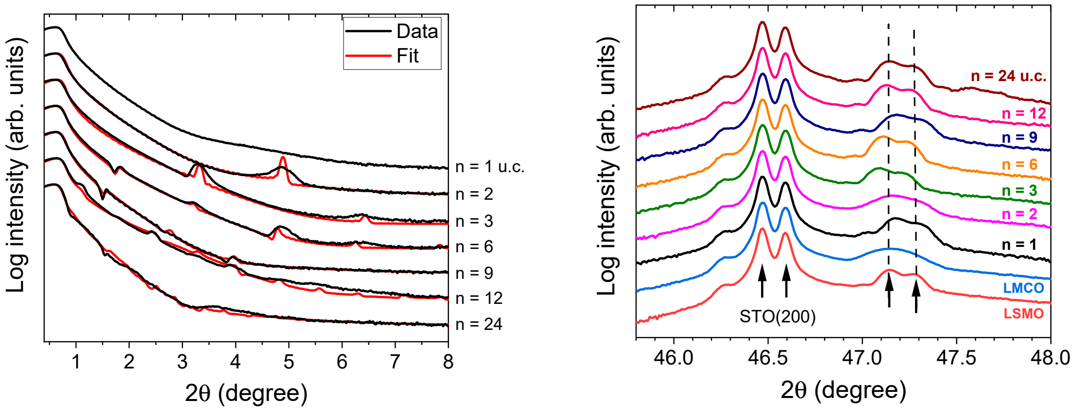

Figure S2 Left: XRR patterns (black curves) and fitting curves using the program *GenX 3* [40] of $[LSMO_n/LMCO_n]_m$/STO(100) SLs revealing superstructure peaks of the bilayers. The fit parameters are listed in Table S1. Right: X-Ray diffraction patterns of the SLs reveal a very similar out-of-plane lattice parameter $c \approx 3.852\pm0.004$ Å for all SLs and single LSMO and LMCO films. The arrows indicate the $k_{\alpha1}/k_{\alpha2}$ spliting of the STO(200) peak and of the (002) peak of most SLs.

[40] A. Glavic and M. Björck, “GenX *3*: the latest generation of an established tool”, J. Appl. Cryst. 55, 1063 (2022)

Table S1 *GenX* 3 fit parameters for $[LSMO_n/LMCO_n]_m$/STO(100) SLs

n (u.c.)	Targeted bilayer thickness d_{2n} (nm)	Measured d_{2n} (nm)	Deviation per bilayer	Average interfacial roughness σ_{RMS} (nm)
2	1.54	1.85	+ 1 u.c.	0.4
3	2.31	2.75	+ 1 u.c.	0.5
6	4.62	5.68	+ 3 u.c.	0.5
9	6.93	7.06	< 1 u.c.	0.9
12	9.24	11.5	+ 6 u.c.	0.6
24	18.49	21.1	+ 7 u.c.	1.1

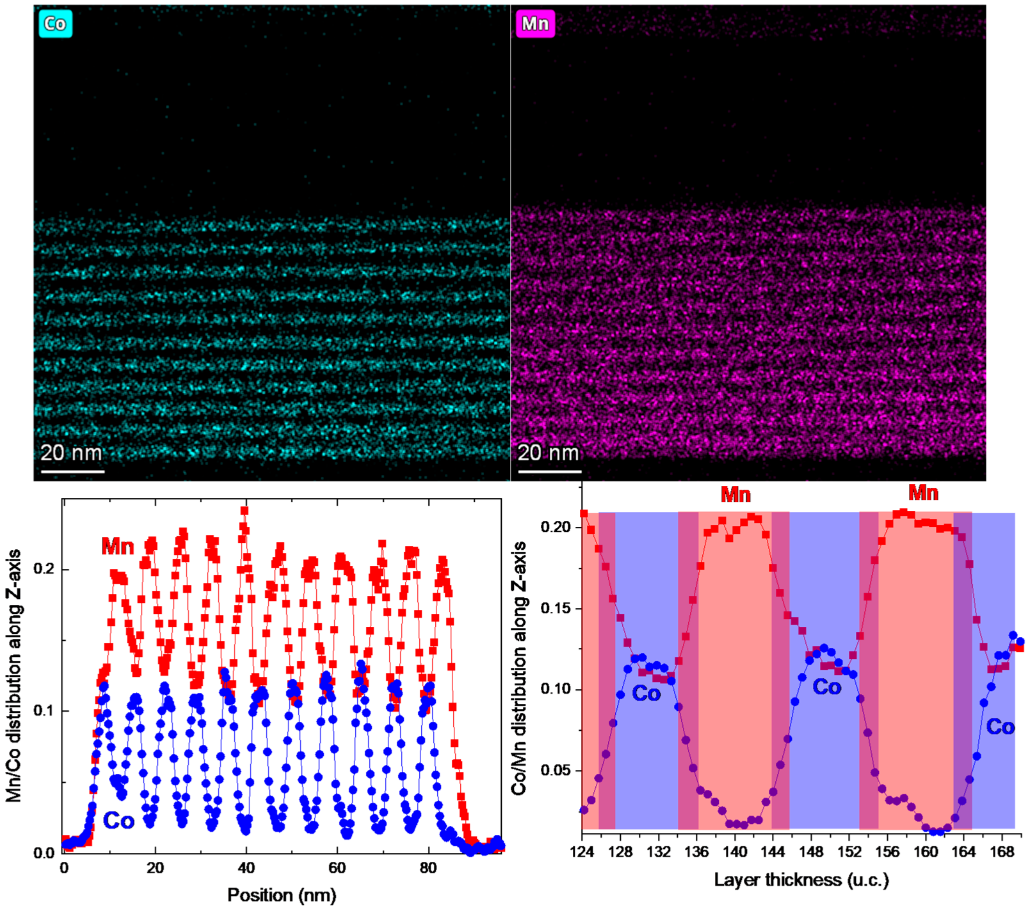

Figure S3 Distribution of Co (top, left) and Mn (top, right) atoms within the $[(LMCO)_9(LSMO)_9]_{11}$ SL obtained by using energy dispersive X-ray microanalysis (EDX) (left panels) shows a clear chemical contrast between the LMCO and the LSMO layers. A linear scan along the growth direction of the whole SL (bottom, left) and along the selected layers in the middle of SL (bottom, right), from which a (Co/Mn) intermixing at the LSMO/LMCO interfaces with a thickness ~2 u.c. can be deduced.

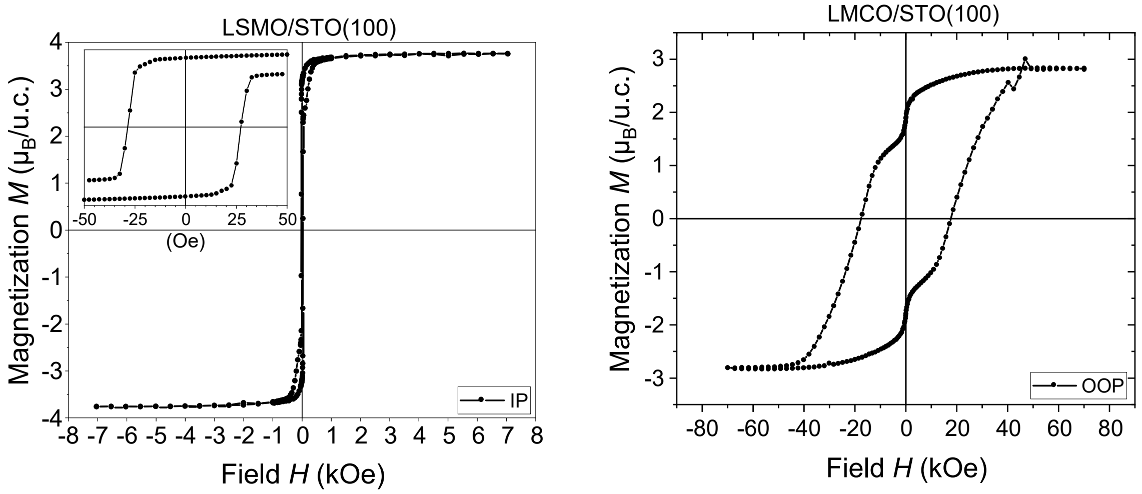

Figure S4: Magnetic hysteresis loops for LSMO/STO(100) (left) and LMCO/STO(100) (right) at 5K along their respective easy axis.

Table S2: Saturation magnetization and coercive field of LSMO/STO(100) and LMCO/STO(100) along their easy axis.

	M_{sat} [μ_B/u.c.]	H_c [Oe]
LSMO/STO(100) IP	3.8(1) (theory: 3.7)	30(10)
LMCO/STO(100) OOP	2.8(1) (theory: 3)	17600(100)

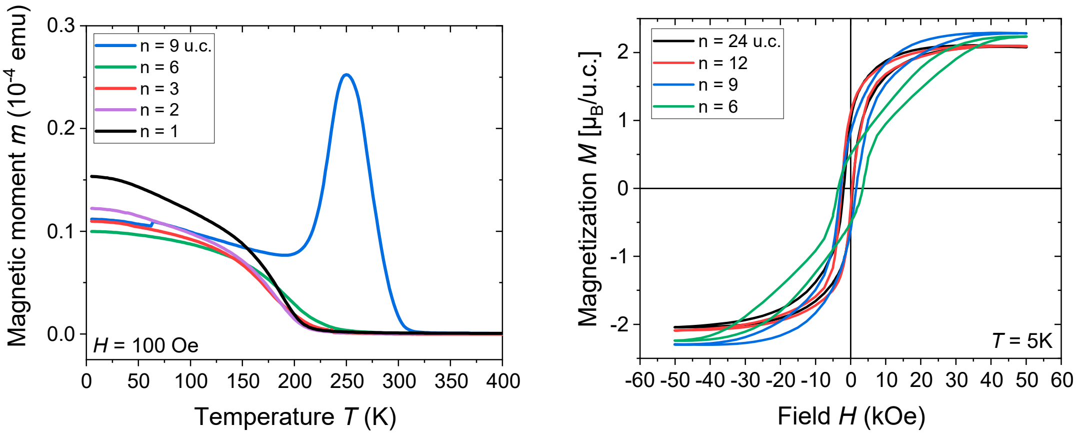

Figure S5 Left: Field-cooled IP magnetic moment of the $[(LMCO)_n/(LSMO_n)]_m$/STO(100) (n = 1-9 u.c.) superlattices at low magnetic field. Spin reorientation transition is missing in SLs with very thin LSMO and LMCO layers. Right: IP Magnetic hysteresis loops of selected $[(LMCO)_n/(LSMO_n)]_m$/STO(100) superlattices.

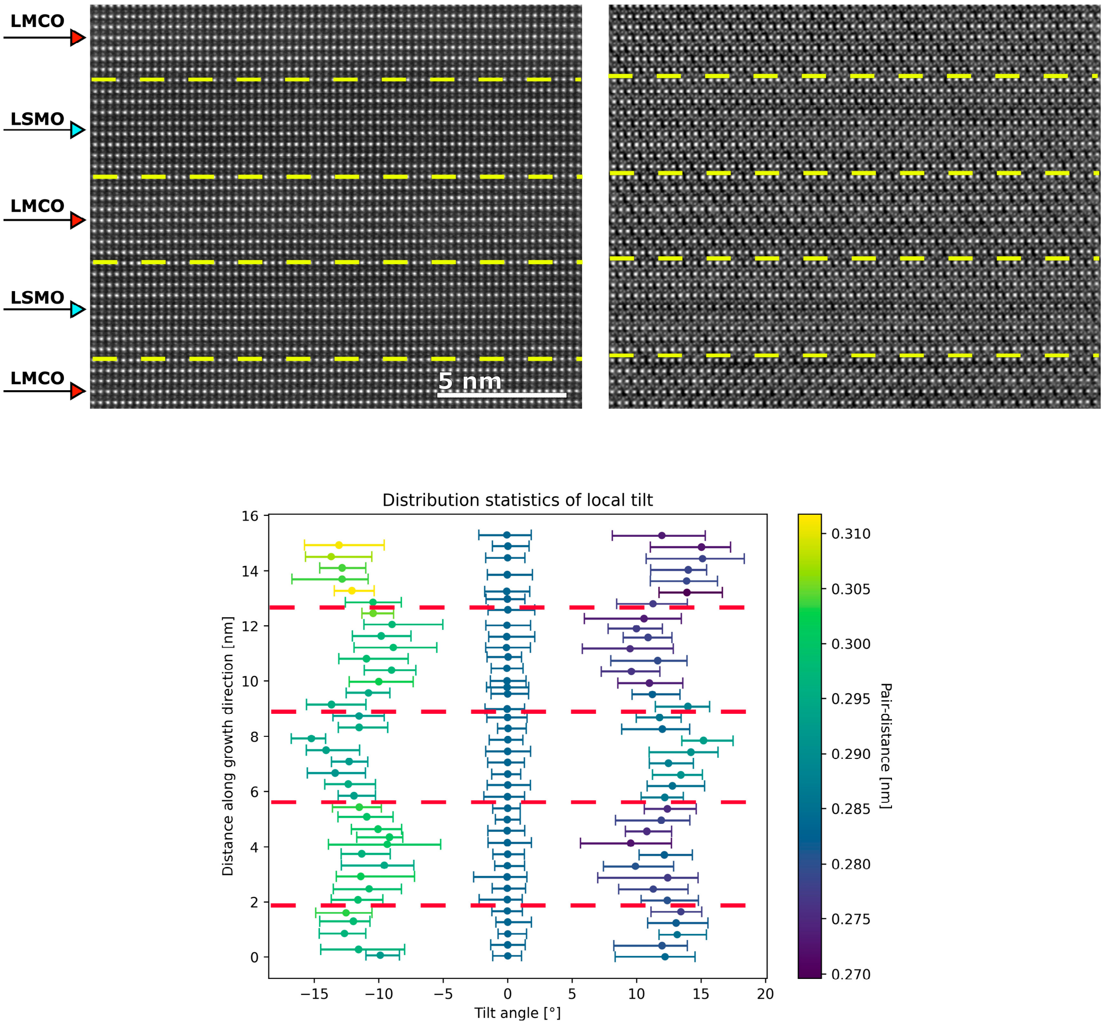

Figure S6 ADF-STEM (top, left), iDPC (top, right) images of the $[LSMO_9/LMCO_9]_{11}/STO(100)$ superlattice, with the corresponding evaluation of octahedral tilt angles θ within the image plane (bottom). Connection to the Mn-O-Mn(Co) angle in the main text is done via $\varphi_{B\text{-}O\text{-}B} = 180° - 2\theta$. The central cluster around θ = 0° (on the right panel) corresponds to the Mn-Mn pairs, while the datapoints around θ = ± 12° correspond to the Mn-O-Mn(Co) bond chains. The analysis reveals continuous change of the octahedral tilt angle at the interfaces between LMCO ($\theta_{LMCO} \approx 13°$) and LSMO ($\theta_{LSMO} \approx 10°$). Error bars indicate the maximum scattering of the angles within one layer, while the standard deviation for all layers lies in the range of σ(θ)=0.5-1.5°.

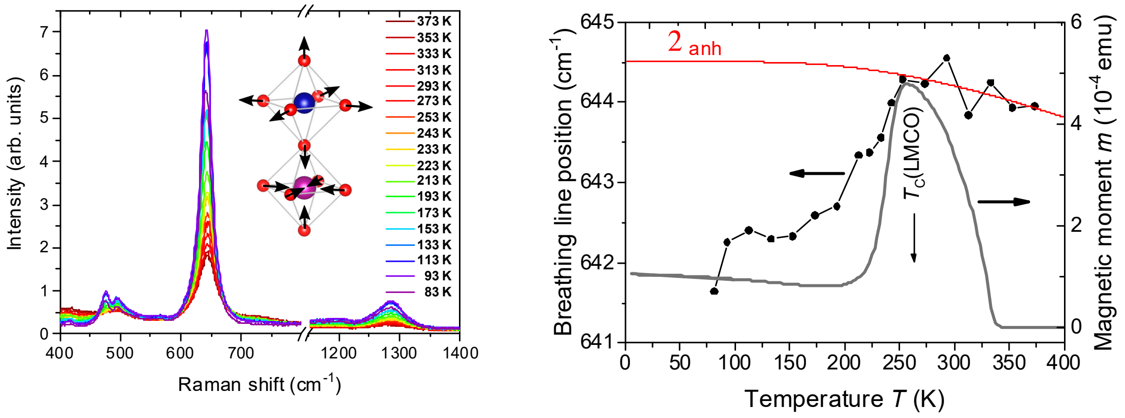

Figure S7 Left: Raman spectra of the n = 24 u.c. SL $[(LSMO)_{24}/(LMCO)_{24}]_4$/STO(100) measured for different temperatures in the range $T \approx$ 100-400 K and an illustration of the 644 cm^{-1} breathing mode vibration; Right: Temperature dependence of the position of the breathing line (left scale) and IP magnetic moment (right scale) indicates a significant increase of the Curie temperature of the LMCO layers within the SL up to about $T_{C,LMCO} \approx$ 260 K. The red line is a fit to the anharmonic line shift $\omega(T) = \omega_0 + C\left(1 + 2/\left(e^{\hbar\omega_0/2k_BT} - 1\right)\right)$ [67] for temperatures $T > T_{C,LMCO}$.

[67] Balkanski, M., Wallis, R. F. & Haro, E. Anharmonic effects in light scattering due to optical phonons in silicon. *Phys. Rev. B* **28,** 1928 (1983).

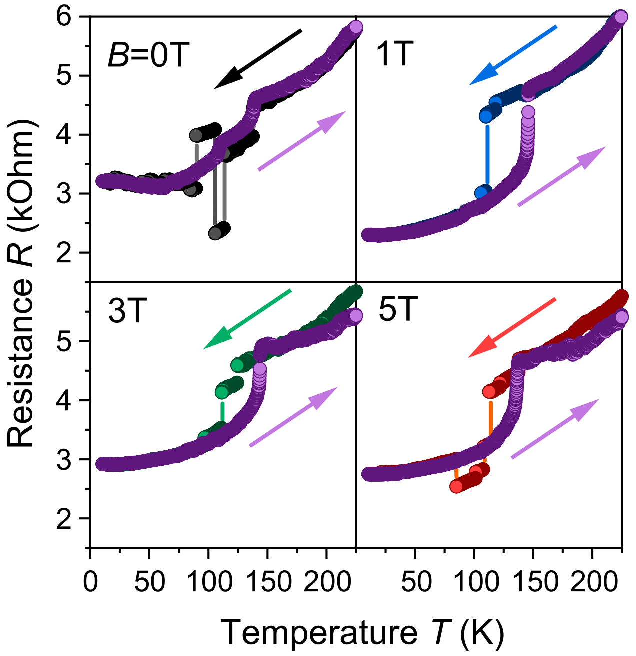

Figure S8 Electrical resistance vs temperature (both current and magnetic field applied in-plane) in the vicinity of structural phase transition in STO at T*=105 K for the $[(LMCO)_9/(LSMO_9)]_{11}$/STO(100) SL at different magnetic fields. The arrows indicate the direction of the temperature scan.

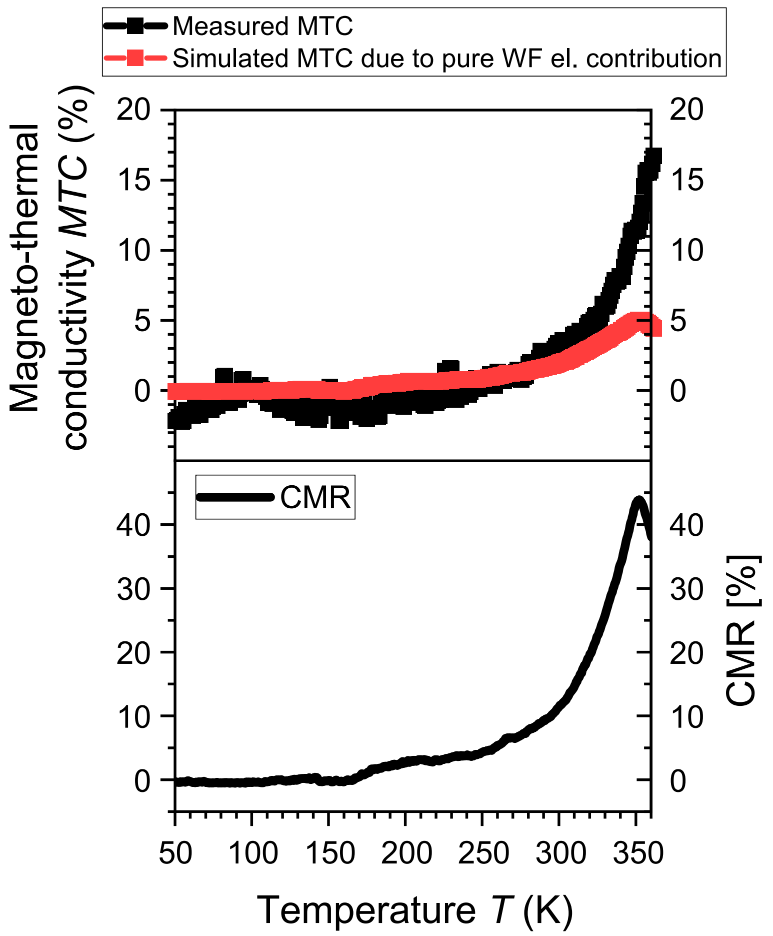

Figure S9 Top panel: measured magneto-thermal conductivity, MTC=100%*[κ(5 T)-κ(0)]/κ(0), (black points) and the estimated from the Wiedemann-Franz (WF) law for electronic contricbution to MTC due to CMR (red points) of the single LSMO/STO(100). Bottom panel: colossal magnetoresistance CMR=100%*[R(0)-R(5 T)]/R(0) of the LSMO/STO(100) thin film shows a typical behaviour with a peak close to T_C≈350 K.
